# Mesmerism

**Published:** 1844-04

**Authors:** 


THE WESTERN JOURNAL.

New Series.—Vol. I.—No. IV.

LOUISVILLE, APRIL 1, 1844.

### MESMERISM.

We think it highly probable, that a few of our readers may feel disposed to quarrel with us for bestowing so much time, and so many pages of our Journal on mesmerism, which, by many, we know, is esteemed an exploded humbug, unworthy of any further investigation. In justification of our course, we must be permitted to say, that to many of the first minds of the age mesmerism does not so appear—that it has found warm advocates among the most intelligent men of every community where experiments have been made in it—that it has been thought worthy of investigation by many learned bodies in our own times, and was examined by a committee of the French Academy, in the last century, at the head of which was Franklin. A subject, said to present so many strange and wonderful phenomena, is assuredly entitled to the consideration of the philosopher. And as a physiological subject it has peculiar claims to the attention of medical men. If not examined by them, who will investigate it carefully, so as to separate all that is false in it from what may be true? The report contained in our last number will appear tedious to most readers; but it presents a mass of testimony which was collected with great care, and which in the minds of the unprejudiced must go a good way towards settling one of the disputed points in mesmerism.

The article in the present number, by Professor Drake, is a commentary upon the facts set forth in the report. We need not bespeak for it the attentive perusal of our readers. No one will fail to read that, and we venture to say that no one will rise from the perusal of it without a strong conviction of the plausibility of the hypothesis advanced. If Dr. Drake has not explained the phenomena attending mesmeric somniloquism, he has offered a theory which strikes us as coming wonderfully near to a satisfactory solution. At all events, it will not be denied that he has produced a very able and interesting paper on the subject, and with it we dismiss mesmerism for the present. Y.

